# S14-5 Girls Active and Change4Life Sports Club - two effective programmes to increase physical activity in schools and colleges

**DOI:** 10.1093/eurpub/ckad133.071

**Published:** 2023-09-11

**Authors:** Chris Wright, Wendy Taylor

**Affiliations:** Youth Sport Trust, England; Youth Sport Trust, England

## Abstract

**Purpose:**

Girls Active aims to tackle declining participation in physical activity of girls aged 10 to 18 and its associated implications on health, wellbeing and education. Change4Life Sports Clubs are designed to address inactivity in 7 to 11 year olds and improve healthy lifestyle behaviours with exercise, food and hydration. Both projects aim to reduce physical inactivity of those children suffering the greatest challenges to achieving the recommended daily physical activity level of an average of 60 minutes of moderate to vigorous physical activity every day.

**Project description:**

Both programmes were developed from evidence reviews and principles that work to engage, inspire and motivate targeted groups of children to be active. They were both developed by the children’s charity Youth Sport Trust, who have used sport and play to improve the lives of children and young people since 1995.

They were co-designed with children and implemented through a national network of 450 School Games Organisers. Youth Sports Trust’s ‘content, delivery, structure’ model was used. For Girls Active we used the Active Lives Children Data from Sport England and Change4Life Sports Clubs were aligned to the Department of Health’s National Child Measurement Programme data to target where the programmes were most needed and to ensure that the right groups of children and young people were included in the interventions.

Both programmes have been externally evaluated through academic partners using pre and post survey data, case studies and control elements and achieved high quality evidence scores. Both programmes have been scaled through a variety of funding streams including London 2012 Olympic Legacy, Sport England Lottery, Corporate Social Responsibility budgets and local public health funding. Girls Active has been adopted in Wales, Scotland and Northern Ireland.

**Conclusions:**

We will share the evidence-based principles that have made these two approaches highly effective in addressing inactivity in primary school aged children and adolescent girls that can be applied in multiple contexts and will share resources and content that can be accessed following the session.

